# Porcelain Inlays

**Published:** 1896-05

**Authors:** C. J. Essig


ARTICLE III.

### PORCELAIN INLAYS.

BY DR. C. J. ESSIG.

The Downie furnace is a little bit of a thing, used with ordinary gas, and it takes only a few minutes to burn a tooth of this kind, and a very short time to cool it. Quite a number of teeth can be done without a great expenditure of time. I really know of nothing more valuable than it is for preparing special forms and sizes of teeth, and for porcelain inlays.

By passing the teeth over a corundum wheel, then getting the right color from one of the Downie preparations, then burning it, the teeth have all the appearance of teeth that have been especially made. I think that the body furnished with the Downie furnace will adhere perfectly and form a very good union between the enamel of the porcelain tooth and itself.

Dr. Guilford.—I have no hesitation in expressing the belief that porcelain work is the coming work. I recognize this just as I long ago recognized that bridge-work had great possibilities, yet at the same time it has its imperfections and limitations.

I have had no experience in baking porcelain. I have had very much experience, however, with this so-called glass filling introduced in place of porcelain filling. Part of the process in each case is the same, and a few points in regard to the procedure may be out of place.

Having prepared a cavity of this kind with parallel or less than parallel walls, a matrix is made of thin platinum or (preferably) a combination of platinum and gold, No. 60, as made by Williams, of New York. Taking a piece of sufficient size to more than cover the cavity, we dress it into place by means of a lead-pencil with a rubber point or end, having first given it the same shape as the pencil end that you write with. In that way the foil can be pressed around the cavity nicely and spread over the edges.

Another way is to use cotton instead of the rubber point; but a better plan is to place your foil over the cavity, make a little indentation in it, then take a piece of beeswax or hard wax, and by slightly warming that and pressing down into the cavity it spreads the foil in all directions and fits the cavity perfectly without wrinkles or folds anywhere. When this is removed, it is simply held over a lamp and the wax melted or burned out.

I think this is the coming work because it has so many possibilities. The objection to a porcelain filling is that it is somewhat difficult to build up the filling in one of these matrices so that when the foil is removed it can be placed in the cavity and not be too high or too low. In making a porcelain filling, you have to be skilful enough to get it just right to answer the purpose, and that s difficult; but the chief objection is the perishability of the material with which it is set.

A couple of years ago I had a patient in whose mouth it was desired to have a Logan crown placed. The direction of the root was such that I could not get one that would stand properly. I selected one of proper shade and nearest size, ground off all of the enamel on the buccal surface, and gave the crown the desired form. This left the porous body of the crown exposed, but it was then nicely covered with enamel and baked in the furnace, and came out very satisfactorily. The Downie furnace is probably the smallest gas-furnace that we have, but the Custer electric oven is simpler and more desirable where the electric current can be had. The electric oven is scarcely larger than an ordinary vulcanizing flask, and it is hoated by simply turning on a button as you would start an electric light.

Dr. C. V. Kratzer.—I have been doing some work in the line of porcelain inlays in the last two years, very much in the manner described by Dr. Christensen. I

find that in addition to the method given by Dr. Christensen, the method Dr. Guilford mentioned, that of using the rubber end of the pencil, sharpening it down to a point, is a good one, but I use the writing end without being sharpened. It is of great aid in pressing the platinum foil to the edges of the cavity; but I do not confine myself to these two. I use a steel burnisher also. I think I can get a sharper edge by finishing up with this. The foil can be readily burnished down, and will admit of considerable burnishing before it becomes hard enough to leave the edges of the cavity, by its springiness.

This work, I think, is especially useful in incisors and cuspids, where a large gold filling would be unsightly, and for that reason objectionable. I have used it in such, and while at first I was skeptical as to the durability of the cement, I found later that my skepticism was entirely unfounded. Of those put in in that way two years ago, I have seen but a few recently, and there is no perceptible wearing away of the cement. The work is very beautiful when you approach the shade of the natural tooth closely and get a good adaptation; from some little distance it can hardly be noticed at all.

In repairing porcelain teeth, changing the shape, form, and color of the teeth, etc., I find it very useful in my practice. The first use I made of it was in the alteration of a Logan crown. I could find none in my stock to fit. The crowns I had were all too small to cover the root. I remedied the difficulty by enlarging the one which came nearest to fitting so as to proximate the contour of the root, thus making a perfect fit by slight subsequent grinding.

I have also repaired broken gum-section blocks by slightly beveling the broken edges and filling the grooves with body; by very careful manipulation the pieces can be thoroughly reunited so that when replaced on the set the joints will be scarcely perceptible, if at all. I have also reattached pins to old blocks by this means.—*Items of Interest.*

